# Editorial: Methodology for emotion-aware education based on artificial intelligence

**DOI:** 10.3389/frai.2025.1704389

**Published:** 2025-10-02

**Authors:** Rosabel Roig-Vila, Miguel Cazorla, Sébastien Lallé

**Affiliations:** ^1^General Teaching Methods and Specific Teaching Methods, University of Alicante, Alicante, Spain; ^2^Computer Science and Artificial Intelligence, University of Alicante, Alicante, Spain; ^3^Laboratoire d'Informatique de Paris 6, LIP6, CNRS, Sorbonne University, Paris, France

**Keywords:** methodology, emotion, education, artificial intelligence, learning

In recent decades, advances in Artificial Intelligence (AI) have opened up unprecedented horizons in educational research. The ability to recognize, interpret and respond to students' emotions presents education with a crucial challenge: to design methodologies that integrate the affective dimension as a fundamental part of the learning process. With this objective in mind, the Research Topic “*Methodology for emotion-aware education based on artificial intelligence*” was born. Its purpose was to bring together work that explores theoretical approaches, technological applications and empirical evidence on Educational AI linked to emotions, bridging affective computing, pedagogy, and human–computer interaction to foster more responsive and ethical emotion-aware learning environments.

This Research Topic offers a pluralistic overview, both in terms of methods and contexts, which allows for reflection on the advances and challenges that arise when introducing AI systems capable of detecting and responding to emotional states in the educational field. Contributions range from analyzing the social impact of scientific production to applying deep learning models, integrating pedagogical beliefs into the adoption of generative technologies, and designing innovative sentiment analysis models. These contributions also highlight ethical, methodological, and practical challenges in the field.

In particular, Ni and Ni introduced ECO-SAM, an innovative sentiment analysis model that combines self-attention techniques with pre-trained neural networks to improve emotion classification in texts with notable increases in accuracy. Its educational relevance lies in the potential of text analysis systems to interpret interactions on learning platforms, forums, and student social networks. The study also opened the possibility of transferring these techniques to the analysis of written work in school environments, which could enrich formative assessment and identify emotional patterns in students' academic and personal writing.

From another perspective, Govea et al. applied deep reinforcement learning models in hybrid learning environments, developing a system capable of detecting emotions in real time by integrating convolutional and recurrent networks. Using data collected from 500 students through cameras, microphones and biometric sensors, the authors showed significant improvements in emotional detection accuracy and learning personalization. This work invites us to rethink hybrid environments as spaces where AI supports cognition and, at the same time, responds to the emotional dimension. However, it also highlights the urgent need to establish regulatory and pedagogical frameworks to ensure that the pursuit of efficiency does not compromise the privacy and emotional wellbeing of students.

Cabero-Almenara et al. focused on a crucial aspect: teacher acceptance of AI in higher education. The study, involving 425 university professors, used the UTAUT2 model to analyze how pedagogical beliefs shape the willingness to integrate generative AI tools. The results reveal that teachers with constructivist orientations are more willing to incorporate these technologies than those with transmissive approaches. These results emphasize that the adoption of AI does not only depend on technical availability, but also on the pedagogical concepts that guide teaching practice. These findings highlight the need for professional training programs that address the diversity of beliefs and contexts. In this sense, we see that the future of AI in education will not be played out solely in laboratories, but also in the ability of institutions to support their teachers in processes of pedagogical reflection and continuous professional development.

Zhou et al. offered a novel approach by applying Item Response Theory (IRT) from a student state-aware perspective. Their SAD-IRT model incorporates parameters derived from facial expression analysis using advanced deep learning techniques, which allow for the estimation of item ability and difficulty, in addition to being an additional parameter linked to the cognitive-affective state of the students. The study demonstrates that this approach improves predictive capacity compared to traditional IRT models and even allows responses to be anticipated before they occur. Beyond its technical value, the article proposes a paradigm shift in educational assessment: considering students' emotions and states as part of the measurement, moving toward more personalized, sensitive and useful assessment systems that can guide teaching and learning.

Finally, Roda-Segarra et al. conducted a pioneering study that went beyond traditional bibliometric indicators by examining more than 6,000 social impact records across 243 publications. The authors revealed that research on AI and emotions in education has a considerable impact on social media and scientific repositories, although academic impact and social visibility do not always align. These findings prompt reflection on how research reaches communities, and how social networks shape the circulation of knowledge. In addition, the study opens the door to reflection on the role of scientific communication in building trust around the use of AI in education, an essential aspect for building a balanced dialogue between innovation, society and schools.

As we can see, the articles gathered in this Research Topic show that AI-mediated emotion-aware education is not a distant goal, but an active field. From a social perspective, research still faces the challenge of extending its impact beyond the academic sphere and ensuring a true transfer to educational communities. From a pedagogical perspective, it is clear that teachers' beliefs influence the adoption of AI. This requires designing training processes that are sensitive to this diversity. Finally, from a technological perspective, advanced models of deep learning and sentiment analysis open up unprecedented possibilities for creating adaptive environments capable of addressing both student performance and emotional wellbeing.

The research published in this Research Topic shows that the combination of pedagogy, AI and emotion-awareness can transform the way we conceive of teaching and learning in the 21st century. However, the path ahead is not without ethical and practical challenges. Issues such as privacy, transparency, and fairness in personalization processes must be non-negotiable principles when using AI in an educational context. The results presented here show that this is possible, but at the same time, they reveal that there is still a long way to go in terms of academic research.

